# Ethyl 2-benzamido-4,5,6,7-tetra­hydro-1-benzothio­phene-3-carboxyl­ate

**DOI:** 10.1107/S1600536810037761

**Published:** 2010-09-30

**Authors:** Asma Mukhtar, M. Nawaz Tahir, Misbahul Ain Khan, Muhammad Naeem Khan

**Affiliations:** a Institute of Chemistry, University of the Punjab, Lahore, Pakistan; b Department of Physics, University of Sargodha, Sargodha, Pakistan; c Applied Chemistry Research Center, PCSIR Laboratories Complex, Lahore 54600, Pakistan

## Abstract

The mol­ecule of the title compound, C_18_H_19_NO_3_S, adopts an approximately planar conformation: the thio­phene and phenyl rings form a dihedral angle of 8.13 (11)° while the ethyl ester group (r.m.s. deviation = 0.0217 Å) is inclined at 1.25 (14) and 8.61 (13)°, respectively, to the thio­phene and phenyl rings. An intra­molecular N—H⋯O hydrogen bond with an *S*(6) ring motif occurs as well as an intra­molecular S⋯O hypervalent inter­action [S⋯O = 2.7369 (18) Å]. The cyclo­hexene ring adopts a half-chair conformation and is disordered over two positions with site occupation factors of 0.641 (6) and 0.359 (6). In the crystal, inversion dimers linked by pairs of O—H⋯O hydrogen bonds generate *R*_2_^2^(10) loops.

## Related literature

For background on thio­phene derivatives, see: Dupin *et al.* (2002 ▶); Khan & Rolim (1983 ▶); Sabnis *et al.* (1999 ▶). For related structures, see: Harrison *et al.* (2006 ▶); Yathirajan *et al.* (2007 ▶). For graph-set notation, see: Bernstein *et al.* (1995 ▶).
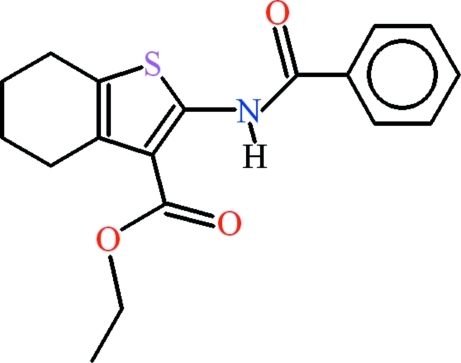


## Experimental

### 

#### Crystal data

C_18_H_19_NO_3_S
*M* *_r_* = 329.40
Monoclinic, 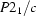

*a* = 8.1061 (2) Å
*b* = 10.6593 (3) Å
*c* = 19.0554 (5) Å
β = 92.500 (1)°
*V* = 1644.92 (8) Å^3^
*Z* = 4
Mo *K*α radiation
μ = 0.21 mm^−1^
*T* = 296 K
0.25 × 0.20 × 0.20 mm

#### Data collection

Bruker Kappa APEXII CCD diffractometer
Absorption correction: multi-scan (*SADABS*; Bruker, 2005 ▶) *T*_min_ = 0.953, *T*_max_ = 0.958
12204 measured reflections
2964 independent reflections
2052 reflections with *I* > 2σ(*I*)
*R*_int_ = 0.037

#### Refinement

*R*[*F* ^2^ > 2σ(*F* ^2^)] = 0.042
*wR*(*F* ^2^) = 0.117
*S* = 1.01
2964 reflections
210 parameters
6 restraints
H-atom parameters constrained
Δρ_max_ = 0.16 e Å^−3^
Δρ_min_ = −0.20 e Å^−3^

### 

Data collection: *APEX2* (Bruker, 2009 ▶); cell refinement: *SAINT* (Bruker, 2009 ▶); data reduction: *SAINT*; program(s) used to solve structure: *SHELXS97* (Sheldrick, 2008 ▶); program(s) used to refine structure: *SHELXL97* (Sheldrick, 2008 ▶); molecular graphics: *ORTEP-3 for Windows* (Farrugia, 1997 ▶) and *PLATON* (Spek, 2009 ▶); software used to prepare material for publication: *WinGX* (Farrugia, 1999 ▶) and *PLATON*.

## Supplementary Material

Crystal structure: contains datablocks text, I. DOI: 10.1107/S1600536810037761/gk2302sup1.cif

Structure factors: contains datablocks I. DOI: 10.1107/S1600536810037761/gk2302Isup2.hkl

Additional supplementary materials:  crystallographic information; 3D view; checkCIF report

## Figures and Tables

**Table 1:** Hydrogen-bond geometry (Å, °)

*D*—H⋯*A*	*D*—H	H⋯*A*	*D*⋯*A*	*D*—H⋯*A*
N1—H1⋯O2	0.86	2.02	2.664 (2)	131
C6—H6⋯O1^i^	0.93	2.44	3.242 (3)	145

Symmetry code: (i) 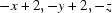.

**Fig. 1.**
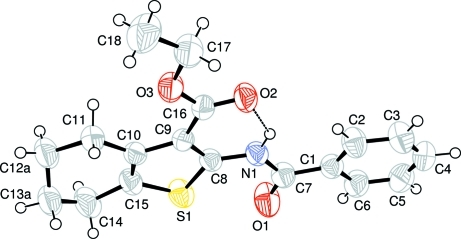
View of the title compound with the atom numbering scheme. The displacement ellipsoids are drawn at the 50% probability level. H atoms are shown as small spheres of arbitrary radii. The dotted line shows intramolecular hydrogen bonding.

## supplementary crystallographic information

### Comment

Thiophenes and their condensed derivatives have been described in the literature (Khan & Rolim, 1983). Tetrahydro-1-benzothiophenes are intermediates for many useful products such as dyes and pharmaceuticals (Sabnis *et al.*, 1999) and compounds containing thrombolytic activity (Dupin *et al.*, 2002). The title compound (Fig. 1) has been prepared for the derivatization of other compounds.

The crystal structures of  ethyl 2-acetylamino-4,5,6,7-tetrahydro-1-benzothiophene-3-carboxylate (Harrison *et al.*, 2006) and  *i.e.* ethyl 2-(propionylamino)-4,5,6,7-tetrahydro-1-benzothiophene-3-carboxylate (Yathirajan *et al.*, 2007) have been published which are related to the title compound.

In the title compound, the heterocyclic ring A (S1/C8/C9/C10/C15) and the phenyl ring B (C1—C6) are planar.  The dihedral angle between A/B is 8.13 (11)°. The ethyl ester group C (O2/C16/O3/C17/C18) is planar with r. m. s. deviation of 0.0217 Å and is inclined at 1.25 (14) and 8.61 (13)° with the rings A and B, respectively. In the title compound an S(6) ring motif (Bernstein *et al.*, 1995) is formed due to intramolecular H-bonding of N—H···O type (Table 1, Fig. 1).  Two methylene groups of the cyclohexene ring in half-chair conformation are disordered over two positions with site occupation factors of 0.641 (6) and 0.359 (6).

### Experimental

Ethyl 2-amino-4,5,6,7-tetrahydro-1-benzothiophene-3-carboxylate (0.3 g, 1.0 mmol) was dissolved in 10 ml of chloroform. To this solution 0.15 ml of benzoyl chloride was added and heated under reflux for 9 h. The solvent was removed and the residue was recrystallized from ethanol to affoard the colorless prism.

### Refinement

In the cyclohexene ring  two methylene groups  are disordered over two positions with site occupation factors of 0.641 (6) and 0.359 (6). The disordered C atoms were refined anisotropically with equal displacement parameters (EADP instruction of SHELXL97).

The H atoms were positioned geometrically (N—H = 0.86, C–H = 0.93–0.97 Å) and refined as riding with *U*_iso_(H) = *xU*_eq_(C, N), where *x* = 1.5 for methyl and *x* = 1.2 for other H atoms.

### Crystal data

**Table:**

C_18_H_19_NO_3_S	*F*(000) = 696
*M**_r_* = 329.40	*D*_x_ = 1.330 Mg m^−^^3^
Monoclinic, *P*2_1_/*c*	Mo *K*α radiation, λ = 0.71073 Å
Hall symbol: -P 2ybc	Cell parameters from 2052 reflections
*a* = 8.1061 (2) Å	θ = 2.5–25.3°
*b* = 10.6593 (3) Å	µ = 0.21 mm^−^^1^
*c* = 19.0554 (5) Å	*T* = 296 K
β = 92.500 (1)°	Prism, colorless
*V* = 1644.92 (8) Å^3^	0.25 × 0.20 × 0.20 mm
*Z* = 4	

### Data collection

**Table:**

Bruker Kappa APEXII CCD diffractometer	2964 independent reflections
Radiation source: fine-focus sealed tube	2052 reflections with *I* > 2σ(*I*)
graphite	*R*_int_ = 0.037
Detector resolution: 8.10 pixels mm^-1^	θ_max_ = 25.3°, θ_min_ = 2.5°
ω scans	*h* = −9→9
Absorption correction: multi-scan (*SADABS*; Bruker, 2005)	*k* = −12→12
*T*_min_ = 0.953, *T*_max_ = 0.958	*l* = −22→22
12204 measured reflections	

### Refinement

**Table:**

Refinement on *F*^2^	Primary atom site location: structure-invariant direct methods
Least-squares matrix: full	Secondary atom site location: difference Fourier map
*R*[*F*^2^ > 2σ(*F*^2^)] = 0.042	Hydrogen site location: inferred from neighbouring sites
*wR*(*F*^2^) = 0.117	H-atom parameters constrained
*S* = 1.01	*w* = 1/[σ^2^(*F*_o_^2^) + (0.0503*P*)^2^ + 0.4332*P*] where *P* = (*F*_o_^2^ + 2*F*_c_^2^)/3
2964 reflections	(Δ/σ)_max_ < 0.001
210 parameters	Δρ_max_ = 0.16 e Å^−^^3^
6 restraints	Δρ_min_ = −0.19 e Å^−^^3^

### Special details

**Table:**

Geometry. Bond distances, angles *etc*. have been calculated using the rounded fractional coordinates. All su's are estimated from the variances of the (full) variance-covariance matrix. The cell e.s.d.'s are taken into account in the estimation of distances, angles and torsion angles
Refinement. Refinement of *F*^2^ against ALL reflections. The weighted *R*-factor *wR* and goodness of fit *S* are based on *F*^2^, conventional *R*-factors *R* are based on *F*, with *F* set to zero for negative *F*^2^. The threshold expression of *F*^2^ > σ(*F*^2^) is used only for calculating *R*-factors(gt) *etc*. and is not relevant to the choice of reflections for refinement. *R*-factors based on *F*^2^ are statistically about twice as large as those based on *F*, and *R*- factors based on ALL data will be even larger.

### Fractional atomic coordinates and isotropic or equivalent isotropic displacement parameters (Å^2^)

**Table:**

	*x*	*y*	*z*	*U*_iso_*/*U*_eq_	Occ. (<1)
S1	0.75739 (8)	0.64947 (6)	0.09851 (3)	0.0685 (2)	
O1	0.9000 (2)	0.82309 (16)	0.01442 (9)	0.0875 (7)	
O2	0.8026 (2)	0.41537 (14)	−0.09850 (7)	0.0658 (6)	
O3	0.6816 (2)	0.26412 (14)	−0.03830 (7)	0.0666 (6)	
N1	0.8625 (2)	0.63457 (16)	−0.03524 (8)	0.0527 (6)	
C1	0.9770 (3)	0.8019 (2)	−0.10411 (11)	0.0545 (7)	
C2	0.9702 (3)	0.7346 (2)	−0.16629 (12)	0.0679 (9)	
C3	1.0262 (3)	0.7864 (3)	−0.22706 (13)	0.0781 (10)	
C4	1.0909 (3)	0.9047 (3)	−0.22675 (14)	0.0804 (11)	
C5	1.1013 (3)	0.9711 (3)	−0.16569 (16)	0.0830 (11)	
C6	1.0438 (3)	0.9216 (2)	−0.10479 (13)	0.0697 (9)	
C7	0.9121 (3)	0.7563 (2)	−0.03689 (12)	0.0582 (8)	
C8	0.7865 (2)	0.5770 (2)	0.01970 (10)	0.0484 (7)	
C9	0.7256 (2)	0.45660 (19)	0.01795 (10)	0.0464 (7)	
C10	0.6520 (2)	0.4227 (2)	0.08292 (10)	0.0505 (7)	
C11	0.5695 (3)	0.2999 (2)	0.09967 (11)	0.0615 (8)	
C12A	0.5302 (8)	0.2864 (5)	0.1768 (3)	0.0771 (13)	0.641 (6)
C13A	0.4684 (7)	0.4071 (4)	0.2069 (3)	0.0771 (13)	0.641 (6)
C14	0.5952 (3)	0.5146 (3)	0.20270 (12)	0.0820 (10)	
C15	0.6620 (3)	0.5167 (2)	0.12986 (11)	0.0601 (8)	
C16	0.7408 (3)	0.3798 (2)	−0.04470 (11)	0.0518 (7)	
C17	0.6953 (4)	0.1819 (2)	−0.09806 (13)	0.0804 (10)	
C18	0.6130 (4)	0.0615 (3)	−0.08052 (16)	0.1017 (13)	
C12B	0.4639 (11)	0.3191 (11)	0.1642 (4)	0.0771 (13)	0.359 (6)
C13B	0.5564 (13)	0.3883 (8)	0.2231 (4)	0.0771 (13)	0.359 (6)
H1	0.88006	0.58943	−0.07157	0.0632*	
H4	1.12771	0.93963	−0.26797	0.0964*	
H2	0.92751	0.65362	−0.16705	0.0815*	
H3	1.01981	0.74051	−0.26860	0.0936*	
H11A	0.64111	0.23142	0.08692	0.0738*	
H11B	0.46774	0.29269	0.07120	0.0738*	
H12A	0.44709	0.22178	0.18136	0.0926*	0.641 (6)
H12B	0.62887	0.26010	0.20338	0.0926*	0.641 (6)
H13A	0.44302	0.39369	0.25557	0.0926*	0.641 (6)
H13B	0.36703	0.43138	0.18147	0.0926*	0.641 (6)
H14A	0.54276	0.59409	0.21247	0.0985*	
H14B	0.68476	0.50168	0.23738	0.0985*	
H17A	0.81052	0.16736	−0.10724	0.0962*	
H17B	0.64183	0.21915	−0.13955	0.0962*	
H18A	0.66559	0.02642	−0.03888	0.1525*	
H18B	0.62180	0.00366	−0.11881	0.1525*	
H18C	0.49861	0.07682	−0.07259	0.1525*	
H5	1.14776	1.05084	−0.16517	0.0996*	
H6	1.04969	0.96880	−0.06371	0.0837*	
H12C	0.36549	0.36616	0.15021	0.0926*	0.359 (6)
H12D	0.42929	0.23798	0.18130	0.0926*	0.359 (6)
H13C	0.48949	0.39036	0.26402	0.0926*	0.359 (6)
H13D	0.65770	0.34371	0.23564	0.0926*	0.359 (6)

### Atomic displacement parameters (Å^2^)

**Table:**

	*U*^11^	*U*^22^	*U*^33^	*U*^12^	*U*^13^	*U*^23^
S1	0.0836 (4)	0.0704 (4)	0.0522 (3)	−0.0085 (3)	0.0127 (3)	−0.0147 (3)
O1	0.1332 (16)	0.0676 (12)	0.0634 (10)	−0.0298 (11)	0.0243 (10)	−0.0167 (9)
O2	0.0950 (11)	0.0567 (10)	0.0472 (9)	−0.0046 (8)	0.0196 (8)	−0.0014 (7)
O3	0.0983 (12)	0.0495 (9)	0.0532 (9)	−0.0073 (8)	0.0168 (8)	−0.0036 (7)
N1	0.0610 (11)	0.0512 (11)	0.0463 (10)	−0.0008 (8)	0.0086 (8)	−0.0027 (8)
C1	0.0553 (12)	0.0529 (13)	0.0555 (13)	−0.0032 (10)	0.0045 (10)	0.0017 (11)
C2	0.0746 (15)	0.0689 (16)	0.0609 (14)	−0.0185 (12)	0.0098 (12)	−0.0028 (13)
C3	0.0868 (18)	0.089 (2)	0.0591 (15)	−0.0080 (15)	0.0115 (13)	−0.0011 (14)
C4	0.0936 (19)	0.0776 (19)	0.0714 (17)	0.0040 (15)	0.0196 (14)	0.0237 (16)
C5	0.101 (2)	0.0565 (16)	0.093 (2)	−0.0055 (14)	0.0221 (16)	0.0151 (15)
C6	0.0878 (17)	0.0550 (15)	0.0670 (15)	−0.0053 (12)	0.0108 (13)	0.0003 (12)
C7	0.0642 (14)	0.0529 (14)	0.0576 (13)	−0.0069 (11)	0.0053 (10)	−0.0027 (12)
C8	0.0476 (11)	0.0559 (13)	0.0418 (11)	0.0043 (10)	0.0031 (9)	−0.0007 (9)
C9	0.0489 (11)	0.0482 (12)	0.0420 (11)	0.0049 (9)	0.0025 (9)	0.0029 (9)
C10	0.0485 (12)	0.0593 (14)	0.0439 (11)	0.0080 (10)	0.0039 (9)	0.0047 (10)
C11	0.0621 (13)	0.0656 (15)	0.0576 (13)	0.0034 (11)	0.0117 (10)	0.0105 (11)
C12A	0.076 (3)	0.098 (2)	0.0587 (18)	0.004 (2)	0.0198 (18)	0.0171 (15)
C13A	0.076 (3)	0.098 (2)	0.0587 (18)	0.004 (2)	0.0198 (18)	0.0171 (15)
C14	0.0943 (18)	0.105 (2)	0.0479 (13)	0.0045 (16)	0.0172 (12)	−0.0045 (14)
C15	0.0626 (14)	0.0730 (16)	0.0452 (12)	0.0009 (11)	0.0096 (10)	−0.0011 (11)
C16	0.0594 (13)	0.0505 (13)	0.0456 (12)	0.0025 (10)	0.0044 (10)	0.0043 (10)
C17	0.127 (2)	0.0565 (15)	0.0587 (15)	−0.0111 (15)	0.0169 (14)	−0.0116 (12)
C18	0.154 (3)	0.0614 (18)	0.090 (2)	−0.0203 (18)	0.0097 (19)	−0.0084 (16)
C12B	0.076 (3)	0.098 (2)	0.0587 (18)	0.004 (2)	0.0198 (18)	0.0171 (15)
C13B	0.076 (3)	0.098 (2)	0.0587 (18)	0.004 (2)	0.0198 (18)	0.0171 (15)

### Geometric parameters (Å, °)

**Table:**

S1—C8	1.714 (2)	C13B—C14	1.440 (9)
S1—C15	1.732 (2)	C14—C15	1.512 (3)
O1—C7	1.217 (3)	C17—C18	1.491 (4)
O2—C16	1.221 (3)	C2—H2	0.9300
O3—C16	1.331 (3)	C3—H3	0.9300
O3—C17	1.445 (3)	C4—H4	0.9300
N1—C7	1.359 (3)	C5—H5	0.9300
N1—C8	1.381 (2)	C6—H6	0.9300
N1—H1	0.8600	C11—H11A	0.9700
C1—C2	1.384 (3)	C11—H11B	0.9700
C1—C6	1.386 (3)	C12A—H12A	0.9700
C1—C7	1.488 (3)	C12A—H12B	0.9700
C2—C3	1.378 (3)	C12B—H12D	0.9700
C3—C4	1.366 (4)	C12B—H12C	0.9700
C4—C5	1.361 (4)	C13A—H13B	0.9700
C5—C6	1.375 (4)	C13A—H13A	0.9700
C8—C9	1.375 (3)	C13B—H13C	0.9700
C9—C10	1.444 (3)	C13B—H13D	0.9700
C9—C16	1.457 (3)	C14—H14A	0.9700
C10—C15	1.343 (3)	C14—H14B	0.9700
C10—C11	1.510 (3)	C17—H17A	0.9700
C11—C12A	1.524 (6)	C17—H17B	0.9700
C11—C12B	1.542 (8)	C18—H18B	0.9600
C12A—C13A	1.503 (7)	C18—H18C	0.9600
C12B—C13B	1.514 (12)	C18—H18A	0.9600
C13A—C14	1.544 (6)		
			
C8—S1—C15	90.83 (10)	C1—C6—H6	120.00
C16—O3—C17	116.70 (17)	C5—C6—H6	120.00
C7—N1—C8	125.72 (18)	C10—C11—H11A	109.00
C7—N1—H1	117.00	C10—C11—H11B	109.00
C8—N1—H1	117.00	C12A—C11—H11A	109.00
C2—C1—C6	118.0 (2)	C12A—C11—H11B	109.00
C2—C1—C7	124.4 (2)	H11A—C11—H11B	108.00
C6—C1—C7	117.5 (2)	C12B—C11—H11A	131.00
C1—C2—C3	120.6 (2)	C12B—C11—H11B	88.00
C2—C3—C4	120.5 (2)	C11—C12A—H12A	109.00
C3—C4—C5	119.6 (3)	C11—C12A—H12B	109.00
C4—C5—C6	120.8 (3)	C13A—C12A—H12A	109.00
C1—C6—C5	120.5 (2)	C13A—C12A—H12B	109.00
O1—C7—C1	123.0 (2)	H12A—C12A—H12B	108.00
O1—C7—N1	120.4 (2)	C13B—C12B—H12C	109.00
N1—C7—C1	116.61 (19)	C11—C12B—H12C	109.00
S1—C8—C9	112.26 (14)	C11—C12B—H12D	109.00
S1—C8—N1	123.14 (16)	C13B—C12B—H12D	109.00
N1—C8—C9	124.60 (18)	H12C—C12B—H12D	108.00
C8—C9—C16	120.08 (17)	C12A—C13A—H13A	109.00
C10—C9—C16	127.95 (18)	C14—C13A—H13A	109.00
C8—C9—C10	111.98 (17)	C14—C13A—H13B	109.00
C11—C10—C15	121.34 (18)	C12A—C13A—H13B	109.00
C9—C10—C11	126.98 (18)	H13A—C13A—H13B	108.00
C9—C10—C15	111.67 (19)	C14—C13B—H13C	109.00
C10—C11—C12B	108.7 (5)	C14—C13B—H13D	109.00
C10—C11—C12A	113.5 (3)	C12B—C13B—H13C	109.00
C11—C12A—C13A	112.0 (4)	C12B—C13B—H13D	109.00
C11—C12B—C13B	112.4 (7)	H13C—C13B—H13D	108.00
C12A—C13A—C14	112.4 (4)	C13A—C14—H14A	110.00
C12B—C13B—C14	111.2 (7)	C13A—C14—H14B	110.00
C13A—C14—C15	108.9 (3)	C15—C14—H14A	110.00
C13B—C14—C15	110.7 (4)	C13B—C14—H14A	131.00
S1—C15—C10	113.26 (16)	C13B—C14—H14B	81.00
S1—C15—C14	120.80 (18)	C15—C14—H14B	110.00
C10—C15—C14	125.9 (2)	H14A—C14—H14B	108.00
O2—C16—C9	124.47 (19)	C18—C17—H17A	110.00
O3—C16—C9	113.69 (18)	O3—C17—H17A	110.00
O2—C16—O3	121.84 (19)	O3—C17—H17B	110.00
O3—C17—C18	107.2 (2)	H17A—C17—H17B	109.00
C1—C2—H2	120.00	C18—C17—H17B	110.00
C3—C2—H2	120.00	C17—C18—H18C	109.00
C2—C3—H3	120.00	C17—C18—H18B	109.00
C4—C3—H3	120.00	H18B—C18—H18C	109.00
C3—C4—H4	120.00	H18A—C18—H18B	109.00
C5—C4—H4	120.00	H18A—C18—H18C	109.00
C4—C5—H5	120.00	C17—C18—H18A	109.00
C6—C5—H5	120.00		
			
C15—S1—C8—N1	179.88 (17)	S1—C8—C9—C10	0.33 (19)
C15—S1—C8—C9	−0.02 (15)	S1—C8—C9—C16	−179.36 (15)
C8—S1—C15—C10	−0.32 (18)	N1—C8—C9—C10	−179.57 (16)
C8—S1—C15—C14	−178.7 (2)	N1—C8—C9—C16	0.8 (3)
C17—O3—C16—O2	0.5 (3)	C8—C9—C10—C11	178.53 (18)
C17—O3—C16—C9	−178.9 (2)	C8—C9—C10—C15	−0.6 (2)
C16—O3—C17—C18	−176.8 (2)	C16—C9—C10—C11	−1.8 (3)
C8—N1—C7—O1	4.4 (3)	C16—C9—C10—C15	179.1 (2)
C8—N1—C7—C1	−174.38 (19)	C8—C9—C16—O2	−1.6 (3)
C7—N1—C8—S1	−5.3 (3)	C8—C9—C16—O3	177.88 (18)
C7—N1—C8—C9	174.55 (19)	C10—C9—C16—O2	178.8 (2)
C6—C1—C2—C3	−0.9 (4)	C10—C9—C16—O3	−1.8 (3)
C7—C1—C2—C3	176.9 (2)	C9—C10—C11—C12A	170.4 (3)
C2—C1—C6—C5	0.0 (4)	C15—C10—C11—C12A	−10.6 (4)
C7—C1—C6—C5	−178.0 (2)	C9—C10—C15—S1	0.6 (2)
C2—C1—C7—O1	−170.1 (2)	C9—C10—C15—C14	178.9 (2)
C2—C1—C7—N1	8.6 (3)	C11—C10—C15—S1	−178.60 (16)
C6—C1—C7—O1	7.7 (4)	C11—C10—C15—C14	−0.3 (3)
C6—C1—C7—N1	−173.6 (2)	C10—C11—C12A—C13A	39.9 (5)
C1—C2—C3—C4	0.7 (4)	C11—C12A—C13A—C14	−59.8 (6)
C2—C3—C4—C5	0.6 (4)	C12A—C13A—C14—C15	46.7 (5)
C3—C4—C5—C6	−1.6 (4)	C13A—C14—C15—S1	160.7 (3)
C4—C5—C6—C1	1.3 (4)	C13A—C14—C15—C10	−17.5 (4)

### Hydrogen-bond geometry (Å, °)

**Table:**

*D*—H···*A*	*D*—H	H···*A*	*D*···*A*	*D*—H···*A*
N1—H1···O2	0.86	2.02	2.664 (2)	131
C6—H6···O1^i^	0.93	2.44	3.242 (3)	145
C11—H11A···O3	0.97	2.45	2.845 (3)	104

Symmetry codes: (i) −*x*+2, −*y*+2, −*z*.
